# Isolation and characterization of genetic variants of *Orthohantavirus hantanense* from clinical cases of HFRS in Jiangxi Province, China

**DOI:** 10.1371/journal.pntd.0012439

**Published:** 2024-09-05

**Authors:** Shiwen Liu, Zhishi Deng, Jianxiong Li, Long Zou, Xiuhui Sun, Xiaoqing Liu, Yong Shi, Shunqiang Huang, Yangbowen Wu, Jinhui Lei, Peipei Liu, Pei Zhang, Ying Xiong, Zhong-er Long

**Affiliations:** 1 Nanchang Key Laboratory of Microbial Resources Exploitation & Utilization from Poyang Lake Wetland, College of Life Sciences, Jiangxi Normal University, Nanchang, Jiangxi, China; 2 Laboratory of Viral Infectious Disease, the Key Laboratory of Important and Emerging Viral Infectious Diseases of Jiangxi Health Commission, Jiangxi Provincial Center for Disease Control and Prevention, Nanchang, Jiangxi, China; 3 Department of Infectious Diseases, Gao′an People′s Hospital, Gao′an, Jiangxi, China; 4 Laboratory Department, Chongren County Center for Disease Prevention and Control, Chongren, Jiangxi, China; 5 Institutional Center for Shared Technologies and Facilities, Wuhan Institute of Virology, Wuhan, Hubei, China; International Atomic Energy Agency, AUSTRIA

## Abstract

**Background:**

Hemorrhagic fever with renal syndrome (HFRS) is a severe public health problem in Jiangxi province, China. Previous studies reported genetic variants of *Orthohantavirus hantanense* (Hantaan virus, HTNV) in rodents in this area. However, the relationship between HTNV variants and human infection needs to be confirmed. This study aimed to identify the HTNV variants in patients and to understand the clinical characteristics of HFRS caused by these variants.

**Methods:**

Samples were collected from hospitalized suspected cases of HFRS during the acute phase. HFRS cases were confirmed using quantitative real-time RT-PCR. Peripheral blood mononuclear cells (PBMC) from patients with HFRS were inoculated into Vero-E6 cells for viral isolation. The genomic sequences of HTNV from patients were obtained by amplicon-based next-generation sequencing. A retrospective analysis was conducted on the clinical characteristics of the patients.

**Results:**

HTNV RNA was detected in 53 of 183 suspected HFRS patients. Thirteen HTNVs were isolated from 32 PBMCs of HFRS cases. Whole genome sequences of 14 HTNVs were obtained, including 13 isolates in cell culture from 13 patients, and one from plasma of the fatal case which was not isolated successfully in cell culture. Genetic analysis revealed that the HTNV sequence from the 14 patients showed significant variations in nucleotide and amino acid to the HTNV strains found in other areas. Fever (100%, 53/53), thrombocytopenia (100%, 53/53), increased serum aspartate aminotransferase (100%, 53/53), and increased lactate dehydrogenase (96.2%, 51/53) were the most common characteristics. Severe acute kidney injury was observed in 13.2% (7/53) of cases. Clinical symptoms, such as pain, petechiae, and gastrointestinal or respiratory symptoms were uncommon.

**Conclusion:**

The HTNV genetic variants cause human infections in Jiangxi. The clinical symptoms of HFRS caused by the HTNV genetic variant during the acute phase are atypical. In addition to renal dysfunction, attention should be paid to the common liver injuries caused by these genetic variants.

## Author summary

HTNV causes severe HFRS in Eastern Asia. Previous studies reported genetic variants or new genotypes of HTNV in animals in South Korea and many parts of China. However, little is known about the clinical characteristics of patients infected by the HTNV variant, because it is difficult to isolate HTNV from patients, and few HTNV variants were confirmed from patients. In this study, we use PBMC from patient to coculture with Vero-E6 cells and a 28-day passage isolation protocol for vial isolation, 13 HTNVs were successfully isolated from patients with a recovery rate of over 40%. We also established an amplicon-based next-generation sequencing method for HTNV whole gene sequencing and whole genome sequences of HTNVs from 14 patients were obtained. Genetic analysis revealed that the complete sequences from the patients showed significant variations in nucleotide and amino acid to the HTNVs found in other areas. Viral isolation and whole genome sequencing confirm that the HTNV variant causes human infection in Jiangxi province. The clinical symptoms of HFRS caused by the HTNV genetic variant during the acute phase are atypical, and laboratory diagnoses are needed. These results provide useful guidance for orthohantaviruses isolation from clinical cases and are important for HFRS diagnosis in clinical practice.

## Introduction

Orthohantaviruses are zoonotic viruses belonging to the *Hantaviridae* family with a global distribution [1]. Orthohantaviruses comprise three negative single-stranded RNA genome segments, namely small (S), medium (M), and large (L), which encode the nucleocapsid protein (NP), glycoprotein precursor (GP), and viral RNA-dependent RNA polymerase, respectively [2]. Rodents are the natural hosts of orthohantaviruses that cause human infection [3]. In rodents, high copy numbers of the viruses (10^8^−10^11^ copies/mL) were often found but did not cause clinical signs of disease [4,5]. Human infections occur via inhaling the aerosolized excreta of infected rodents in endemic areas [1]. Each year, 150,000 to 200,000 cases of orthohantavirus infection are reported worldwide [6]. In human infections, orthohantaviruses can cause serious diseases, including hemorrhagic fever with renal syndrome (HFRS) and hantavirus pulmonary syndrome, the disease severity usually relates to species of orthohantaviruses and viral loads in patients [7,8]. At least 21 of the 35 orthohantavirus species can cause human infection [9]. *Orthohantavirus hantanense* (Hantaan virus, HTNV) is the prototype orthohantavirus hosted by *Apodemus agrarius* (*A*. *agrarius*) [10]. HTNV is often found in East Asia and causes severe HFRS [11]. HFRS is characterized by fever, pain, bleeding, and acute kidney injury (AKI) with a fatality rate of up to 15% [9,12]. Like other orthohantaviruses, HTNV shows significant genetic diversity and geographic clustering of genetic variants [13]. Previous studies reported genetic variants or new genotypes of HTNV in animals in South Korea and many parts of China [14,15]. The emergence of orthohantavirus variants facilitates potential risks to public health safety [16]. However, little is known about the clinical characteristics of patients infected by the HTNV variant, because it is difficult to isolate HTNV from patients, and few HTNV variants were confirmed from patients.

Jiangxi Province, located in southeast China, is one of the hotspots of HFRS, with 8,981 cases reported between 2005 and 2021 [17]. Previous studies showed HTNV variants (including AYW89-15 and GAW48-19) circulating in rodents in Jiangxi [17,18]. However, the relationship between HTNV variants and human infection needs to be confirmed through viral isolation from patients and whole genomic analysis. To date, no HTNV has been isolated from patients in Jiangxi, the genomic characteristics of these viruses are limited, whether the HTNV variants cause human infection is unclear, and the clinical characteristics of HFRS caused by HTNV in this area are not understood.

Isolation and whole genome sequencing are necessary to enhance our understanding of these pathogens [11]. Orthohantaviruses are difficult to isolate from cell culture [10,19,20]. Owing to their slow replication and non-cytopathic nature in cell culture, the recovery rate of orthohantavirus isolates is low [21]. Isolating orthohantaviruses from clinical samples is more challenging than isolating them from rodent samples, because of the ultralow copy number of viral RNA in the former. Next-generation sequencing (NGS) technologies have been widely used for viral whole genome sequencing [22,23]. Various methods have been used to enrich the genomes of viruses of interest, including amplicon NGS and target capture, among these, PCR amplicon-based NGS offers a sensitive approach for sequencing small genomes [15].

This study aimed to identify HTNV variants in patients with HFRS through viral isolation and one-step amplicon-based NGS and to understand the clinical characteristics of HFRS caused by these variants in Jiangxi province.

## Materials and Methods

### Ethics statement

This study involving human participants was approved by the Ethics Committee of Jiangxi Provincial Center for Disease Control and Prevention (2020BBGL73052) and adhered to the Declaration of Helsinki. Samples were collected after obtaining written informed consent.

### Sample collection

Clinical samples were collected during 2020–2022 in a public hospital (Gao′an People′s Hospital) located in Gao′an City (28.4178°N, 115.3753°E), Jiangxi Province, where HTNV is endemic in rodents. Hospitalized patients residing in an orthohantavirus endemic area, who had febrile syndrome within the last 7 days and did not fit the diagnosis of other known fever diseases were included as suspected cases of HFRS. The suspected HFRS case met the diagnostic criteria for HFRS issued by the National Health Commission of the People’s Republic of China (WS 278–2008). Venous blood (3 mL) was collected from each suspected patient in an EDTA tube (5 mL). Blood samples were temporarily stored at 4°C, and sent to the laboratory within 24 hours after collection.

### Plasma and Peripheral blood mononuclear cells (PBMC) separation

The plasma and PBMC were separated from the blood sample immediately after reception. Plasma was separated by centrifugation at 800× g and 4°C for 10 min. According to the manufacturer’s instructions, PBMCs were separated using Ficoll-Hypaque (TBD Science, China). Finally, the PBMCs were resuspended in 1 mL of PBS (Gibco, USA). Plasma samples were used for orthohantaviruses RNA detection and virus isolation. PBMCs were used for virus isolation.

### Specific diagnosis of HFRS cases

HFRS cases were confirmed using a duplex quantitative real-time RT-PCR (RT-qPCR) designed to detect the S segment of HTNV and *Orthohantavirus seoulense* (SEOV) in plasma, with detection limits of 10 RNA copies/μL for each virus [18]. RNA extraction and RT-qPCR were performed as previously described [18]. Briefly, 40 μL of RNAs was obtained from 140 μL of plasma. RNA detection was conducted in duplicate. Home-made plasmid pGEM-T-HTNV and pGEM-T-SEOV were used as the positive control, while nuclease-free water was used as the negative control. The real-time RT-PCR was validated after confirming that the positive control’s cycle threshold (Ct) value fell within the expected range and the negative control was undetermined. Otherwise, the assay was repeated and then considered validated or not.

### Viral isolation and identification

Vero-E6 cells (ATCC: CCL-81) were used for viral isolation. Vero-E6 cells were grown in the minimal essential medium (MEM) (Gibco, USA) containing 10% fetal bovine serum (FBS) (BI, Israel) and passaged once per 7 days at a 1:4 ratio. Plasma or PBMCs were inoculated Vero-E6 cells for viral isolation using a 28-day-passage protocol. For inoculation, 0.8–1.0 ml of plasma or PBMCs were added to the monolayer of Vero-E6 cells in the growing medium (MEM+10% FBS) in a 25 cm^2^ flask (Corning, Germany) in 48 h after the sample collection. The flask was incubated at 37°C with 5% CO_2_ for 28 days until the next passage. During the cultivation, the supernatant was replaced with 5 mL fresh maintaining media (MEM+2% FBS) every 7 days. After 28 days, infected cells were trypsinized and resuspended with 5 mL of maintaining medium, and 1×10^6^ fresh Vero-E6 cells in 5 mL of the maintaining medium were mixed with the suspension. The mixture was divided into two 25 cm^2^ flasks for another 28-day culture. This process was repeated twice to obtain passage 3 isolates. In parallel, a flask of uninfected Vero-E6 cells was passaged as a control. Viruses in the supernatant were measured using RT-qPCR, and infected cells were collected for orthohantavirus nucleoprotein test by immunofluorescence assay (IFA) every 7 days.

IFA was performed as follows: An aliquot of cells was fixed in cold acetone at room temperature for 20 min and air-dried. Fluorescein isothiocyanate-labeled monoclonal antibody specific for HTNV NP from the Department of Microbiology in the Fourth Military Medical University of China was added to the slides, incubated for 45 min at 37°C, washed with PBS three times, blow-dried, and sealed with glycerol [24]. The slides were observed under a fluorescence microscope.

When the infected cells were IFA positive, 0.5 mL of culture supernatant was inoculated to fresh Vero-E6 cells in a 25 cm^2^ flask and then frozen at -80°C for working stock after incubating at 37°C with 5% CO_2_ for 7 days.

The morphology of the HTNV particles was observed using transmission electron microscopy (TEM) in the Institutional Center for Shared Technologies and Facilities of Wuhan Institute of Virology. Vero-E6 cells were infected with 0.5 mL of culture isolate in a 25 cm^2^ flask. Cells were cultured for 10 days, the culture supernatant was discarded, then the cells were fixed with 2.5% glutaraldehyde followed by 1% osmium acid, and then dehydrated by 30% to 100% alcohol. The samples were encapsulated in epoxy resin and then cut into ultrathin sections (60–80 nm) using an ultramicrotome (EM UC7, Leica) on EM grids. The grids were examined using a Tecnai G2 20 Twin electron microscope (FEI Company) at 200 Kv.

### Whole genome sequencing of HTNV

**Fig 1:**
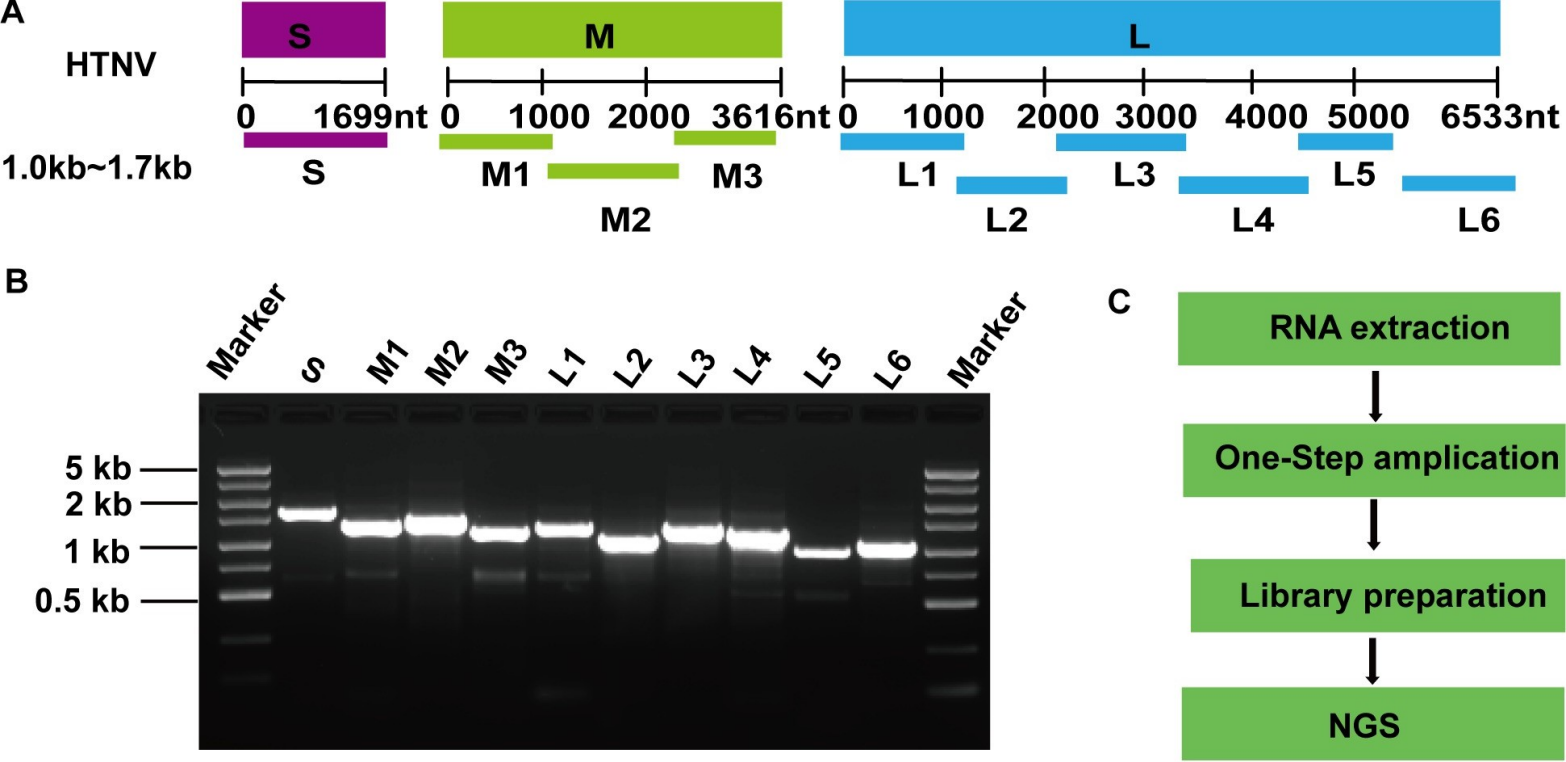
One-step amplicon-based NGS in this study. A. Schematic diagram of HTNV genome covered by amplicons produced by 10 pairs of primers. B. Amplicons obtained using the primers were validated through electrophoresis. C. The workflow of one-step amplicon-based NGS.

To know the genetic characteristics of HTNV from patients, a one-step amplicon-based NGS method was established for whole genome sequencing of HTNV (**Fig 1**). Ten overlapping amplicons were designed using primer pairs based on the alignments of the complete sequences (S, M, and L) of HTNV. Primer sequences are shown in the **S1 Table**.

RNAs from one plasma sample and all the culture isolates were reverse transcribed and amplified using primers and the Superscript III One-step RT-PCR System (Invitrogen, USA) according to the manufacturer’s instructions for target enrichment. The one-step RT-PCR reactions were carried out with the following conditions: 30 min at 50°C, then 2 min at 94°C, this was followed by 30 cycles consisting of 15 s at 94°C, 30 s at 52°C, and 1 min 45 s at 68°C, with a final elongation for 5 min at 68°C. Amplicons of each isolate were pooled into a single mixture for library preparation. The pooled amplicons were purified using AMPure XP Reagent (Beckman Coulter, USA). The libraries were prepared using the Ion Shear Plus Regents Kit, and the Ion Plus Fragment Library Kit (Thermo Fisher, USA) following the manufacturer’s instructions. During the preparation, the libraries for each sample were ligated with unique barcode adapters using the Ion Xpress Barcode Adapters (Thermo Fisher, USA). Libraries were quantified and pooled before generating the template-positive ion sphere particles with Ion 520&530 OT2 reagents (Thermo Fisher, USA). Subsequently, the template-positive ion sphere particles containing the libraries were created with Ion 520&530 OT2 reagents and then loaded onto an Ion 530 chip for sequencing using the Ion GeneStudio S5 System (Thermo Fisher, USA).

The sequencing data was analyzed using CLC Genomics Workbench 21 (Qiagen, Germany). The raw reads were processed by trimming adaptors, primer sequences, and low-quality bases with a phred score threshold of 20. The trimmed sequences were assembled and aligned with the HTNV reference sequences of GAW48-19 (GenBank access number MN985832-MN985834) [18]. A consensus sequence was extracted from the mapping reads for each sample and positions with coverage below 30 were replaced with an ’N’.

### Genetic analysis of HTNV sequences

Alignment was performed using the ClustalW method in MEGA X. Genetic identities were analyzed using BioEdit (Verson7.2.5). Phylogenetic trees were constructed using the complete S, M, and L sequences of the novel HTNVs from 14 patients in this study and 24 published other orthohantaviruses obtained from GenBank (**S2 Table**). Phylogenetic analysis was performed using the maximum likelihood method in the MEGA X software with a bootstrap analysis of 1000 replicates. The gene recombination signal analysis was conducted using RDP5(Version 5.58). Recombination events with *P* values < 0.01, confirmed by four or more methods including the RDP method, GENECONV, MaxChi, Chimaera, BootScan, SiScan, and 3Seq, were identified and classified as instances of recombination [14].

### Clinical characteristic

Clinical symptoms and biological parameters of HFRS cases were retrospectively obtained from electronic medical records in the hospital. Abnormal liver function was defined as any parameter exceeding the upper limit of normal values (ULN) for alanine aminotransferase (ALT), aspartate aminotransferase (AST), lactate dehydrogenase (LDH), γ- glutamyl transferase (GGT), alkaline phosphatase (ALP), or direct bilirubin (DB) [25]. Impaired renal function is characterized by proteinuria, serum creatinine (SCR), or blood urea nitrogen (BUN) levels exceeding the ULN. The SCR level >353.6 μmol/L indicated a severe AKI [26]. Clinical characteristics were described using medians (with an interquartile range) for continuous variables and percentages for categorical variables.

## Result

### Specific diagnosis of HFRS cases

**Fig 2:**
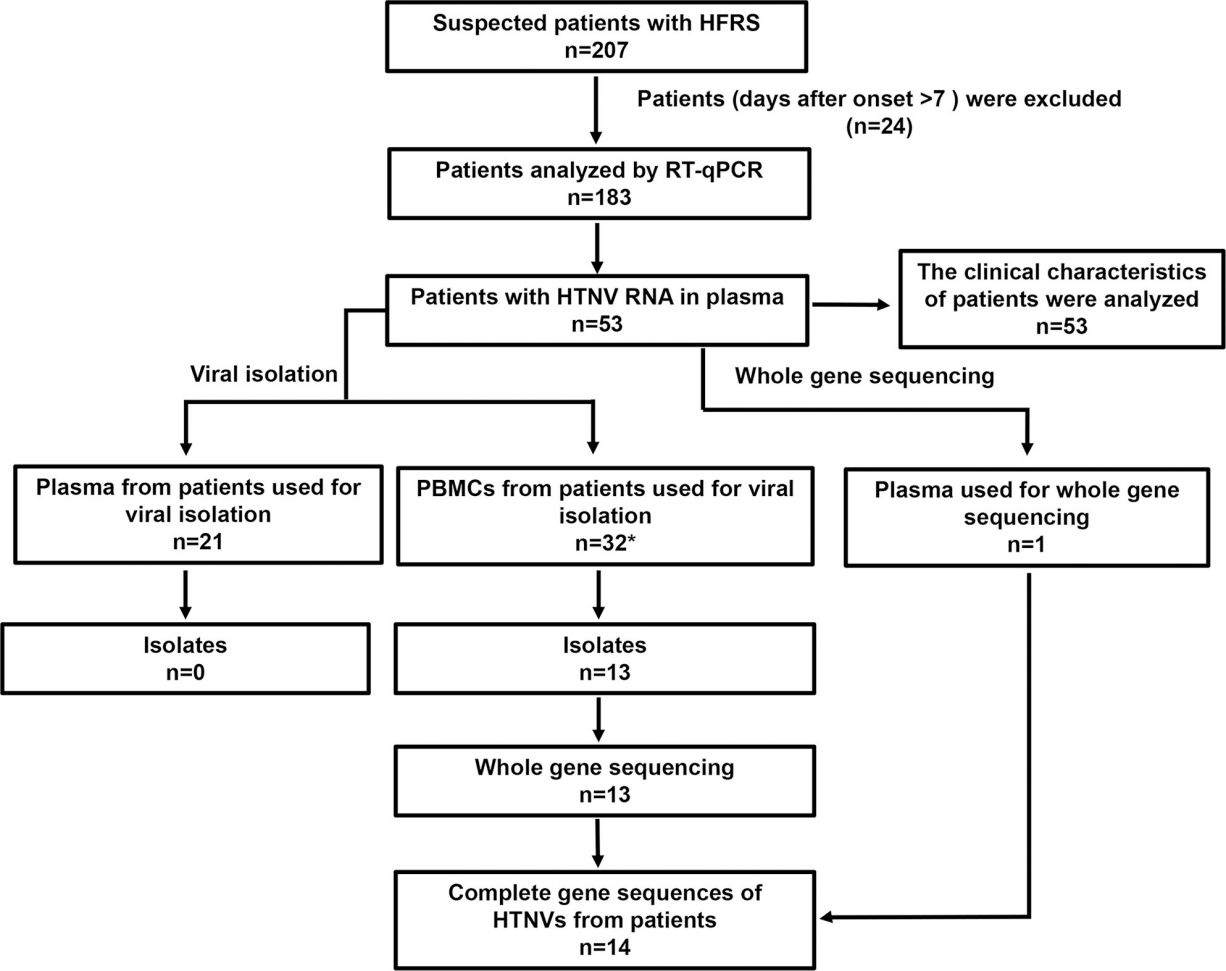
Flow diagram of the study. “*”: Among these 32 patients, virus isolation was performed simultaneously using plasma (isolate n = 0) and PBMCs (isolates n = 3) from 7 paitens, and virus isolation was performed using only PBMC from 25 patients (isolates n = 10).

Among the 207 patients suspected of HFRS in this study, 24 were removed because of missing data, or over 7 days after onset. Blood samples were obtained from 183 suspected cases of HFRS. Plasma samples were separated from blood and analyzed by RT-qPCR, and 53 patients including one patient who died presented HTNV RNA in the plasma (53/183) (**Fig 2**). No SEOV infection was found. The median viral load in the plasma of these patients was 4.2×10^5^ copies RNA/mL.

### Viral isolation

**Fig 3:**
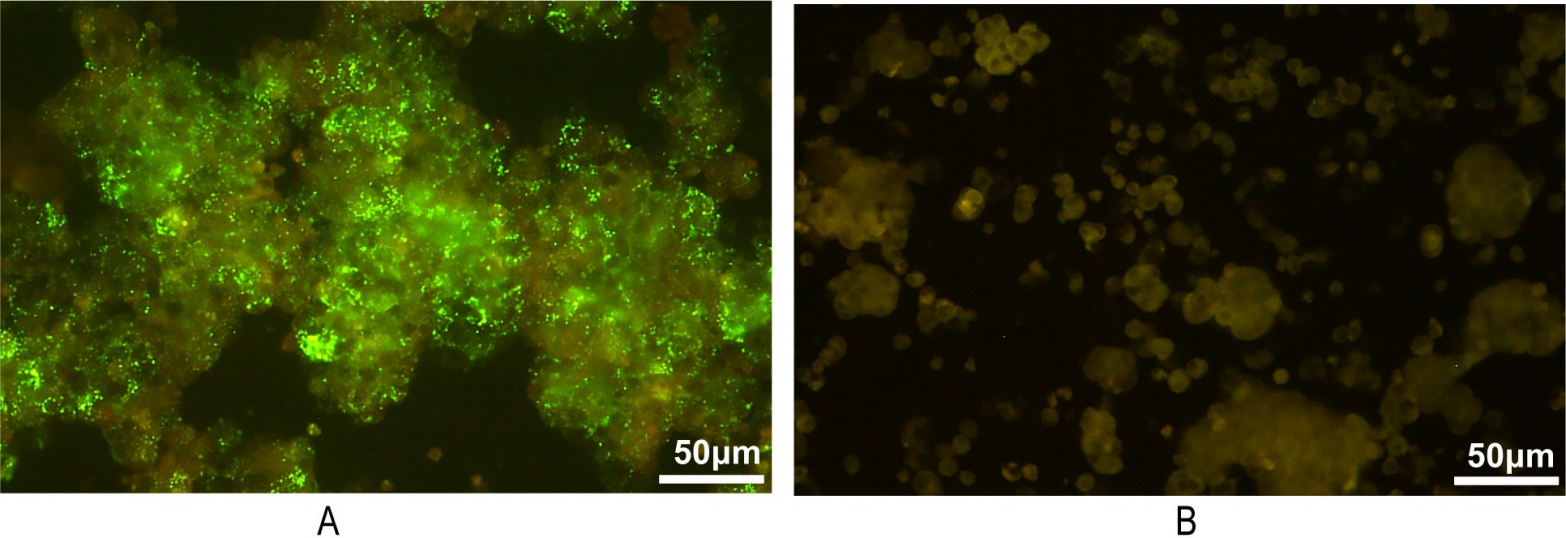
The IFA images of HTNV-infected Vero-E6 cell and Vero-E6 cells control. HTNV-infected Vero-E6 cells show specific bright green fluorescent dots indicating the virus was isolated successfully (A), while the control Vero-E6 cells show no bright green fluorescent dots (B).

To confirm the HTNV genetic variant causes human infection, viruses were tried to isolate from clinical samples in cell culture. Plasma and PBMC from patients were inoculated to Vero-E6 cells to isolate the virus, and 13 HTNV isolates were obtained from PBMCs, with a recovery rate of 40.6% (13/32), no isolates were obtained from plasma samples (n = 21) (**Fig 2**). The infected Vero-E6 cells did not present cytopathic effects, all the isolates were identified as HTNV by RT-qPCR and IFA (**Fig 3**). The virus from clinical samples grew slowly in vitro, and the infected cells were IFA positive at 21–84 days after infection.

**Fig 4:**
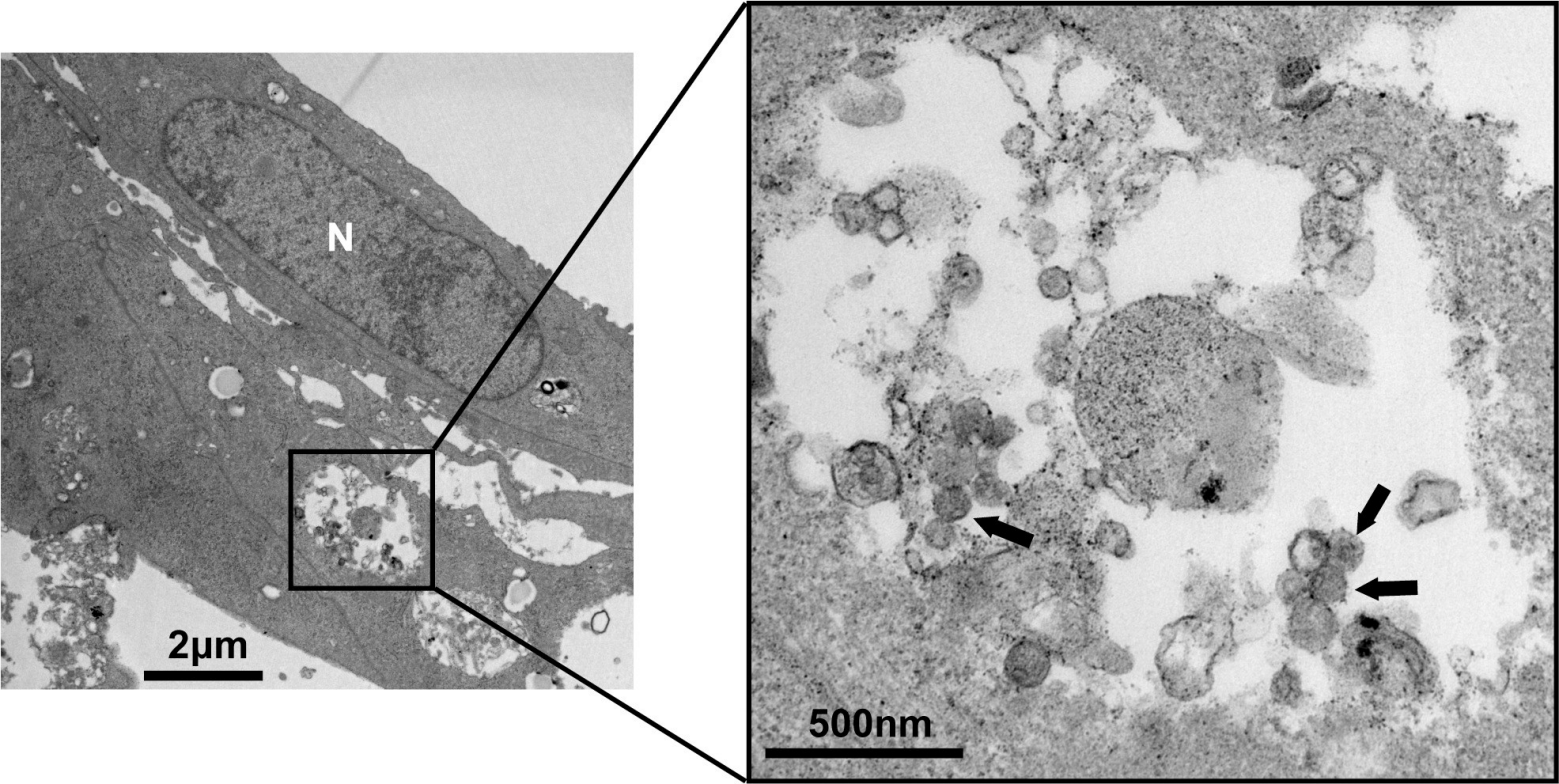
TEM images of HTNV particles (isolate JXGAHu98/2021) in Vero-E6 Cell. A vesicle of the HTNV-infected cell was magnified, virions were observed inside the vesicles, and arrows pointed to the HTNV particles. “N”: the nuclear of the cell.

The TEM images of Vero-E6 cells infected with viral isolates are shown in **Fig 4**. Round virions were observed inside the vesicles of infected cells. The TEM images of virions through the negative stain are shown in **S1 Fig**, the virions were approximately 100–150 nm. The morphology and size of the virions isolated in this study were consistent with HTNV.

### Whole genome sequencing

To know the genetic characteristics of HTNV in patients, a total of 14 samples from 14 patients were sequenced by one-step amplicon-based NGS (**Fig 1**), including13 isolates in cell culture from 13 patients (**Table 1**), one plasma sample from the fatal case (G79) which was not isolated successfully. Raw sequencing data including 14 samples in this study were submitted to the SRA database under the accession number: PRJNA1114415.

**Table 1:** Summary genome sequencing of HTNVs from patients using one-step amplicon-based NGS.

Case number	Isolate	Total reads	Viral reads	The proportion of mapped reads (%)	The average depth of reads			Coverage rate (%)			GenBank Accession number*		
					S	M	L	S	M	L	S	M	L
G80	JXGAHu80/2021	3,894,972	1,884,431	48.4	38,183	25,832	45,577	100.0	100.0	100.0	OL961327	OL961345	OL961364
G86	JXGAHu86/2021	1,623,813	1,234,554	76.0	5,577	7,781	30,085	99.9	100.0	100.0	OL961328	OL961346	OL961365
G88	JXGAHu88/2021	19,592,552	1,925,328	98.3	225,409	349,597	319,297	100.0	99.9	100.0	OL961329	OL961347	OL961366
G97	JXGAHu97/2021	1,446,288	913,591	63.2	1,304	5,314	21,349	99.9	99.9	100.0	OL961330	OL961348	OL961367
G98	JXGAHu98/2021	1,306,953	886,123	67.8	10,807	5,161	16,040	100.0	100.0	100.0	OL961331	OL961349	OL961368
G107	JXGAHu107/2021	777,358	252,320	32.5	3,984	1,745	4,164	100.0	100.0	100.0	OL961332	OL961350	OL961369
G111	JXGAHu111/2021	1,128,187	946,101	83.9	10,391	4,732	17,883	100.0	99.9	100.0	OL961333	OL961351	OL961370
G113	JXGAHu113/2021	777,006	363,776	46.8	1,904	2,352	7,344	100.0	99.9	100.0	OL961334	OL961352	OL961371
G118	JXGAHu118/2021	4,703,504	2,888,717	61.4	41,028	59,277	60,476	100.0	100.0	100.0	OL961335	OL961353	OL961372
G124	JXGAHu124/2021	5,706,312	3,115,320	54.6	47,444	29,928	79,682	100.0	100.0	100.0	OL961336	OL961354	OL961373
G126	JXGAHu126/2021	4,010,589	1,908,969	47.6	22,863	13,176	59,694	100.0	100.0	100.0	OL961337	OL961355	OL961374
G129	JXGAHu129/2021	2,641,004	930,971	35.3	6,503	2,346	32,233	100.0	99.9	100.0	OL961338	OL961356	OL961375
G197	JXGAHu197/2021	2,998,918	2,106,772	70.5	23,607	13,763	46,046	99.8	99.9	100.0	PP540018	PP540019	PP540020
G79	GAHu79/2021	2,477,797	1,120,816	45.7	33,744	9,334	3,009	100.0	100.0	100.0	OL961324	OL961342	OL961361
Average		3,791,804	1,462,699	59.4	33,768	37,881	53,063	100.0	100.0	100.0	/		

*Based on the highly conserved nature of the HTNV terminal sequences, one or two missing bases at both ends of the sequence in some isolates are artificially added when subscribed into the GenBank. The sequences of GAHu79/2021 were recovered from the plasma of the fatal case (G79).

The portion of total viral reads of 14 HTNVs mapped to the reference sequences was 32.5–98.3%. Nearly whole genome sequences of 14 viruses were obtained, with the sequence lengths of S, M, and L being 1695–1699, 3611–3616, and 6532–6533, respectively. The coverage of genomic sequences was 99.8–100%. The average depths of the S, M, and L segments were 33,768, 37,881, and 53,063, respectively (**Table 1**).

### Genetic analysis

Whole genome sequences of HTNVs were analyzed. The nucleotide identities of the complete S, M, and L sequences of the HTNVs in this study were 95.0–99.7%, 95.1–99.8%, and 95.0–99.2%, respectively. The nucleotide identity and deduced amino acid identity to the 76–118 strain, the prototype strain of HTNV, were 84.0–86.7%, and 96.2–97.4% respectively. When aligned to the 76–118 strain, the 14 HTNVs in this study showed at least 2.5% (11/429), 3.1% (35/1135), and 2.0% (43/2151) amino acid mutations in the NP, GP, and viral RNA-dependent RNA polymerase, respectively. The nucleotide identity among the 14 HTNVs in this study and 19 HTNV strains from other areas was 81.1–86.9%.

The HTNVs in this study showed high nucleotide identity (95.0–99.3%) and high amino acids (99.0–99.8%) to the genetic variants strain (GAW48-19) [18] from rodents in Jiangxi Province. The detailed amino acid similarity between the isolates from patients and GAW48-19 is shown in the **S3 Table**. Compared with GAW48-19, each isolate from HFRS patients showed amino acid mutations, these mutations were random, and no consistent amino acid mutation was found.

A comparison analysis of the immune epitope sequences on NP and GP of HTNV from patients in this study and the reference HTNV strains in the **S2 Table** was conducted. Four specific amino acids (L179 on NP, V71, G700, and D967 on GP) related to 6 T cell immune epitopes on NP and GP of HTNVs from Jiangxi province were found. The detailed amino acid mutations in epitopes of HTNVs in Jiangxi province are listed in the **S4 Table.**

The RDP5 analysis detected a recombination event in the L segment in JXGAHu197/2021 by six methods with the *P* values (1.19×10^−29^–6.87×10^−7^) all lower than 0.01 (**S5 Table**), no potential recombination events were detected in other sequences in this study.

**Fig 5:**
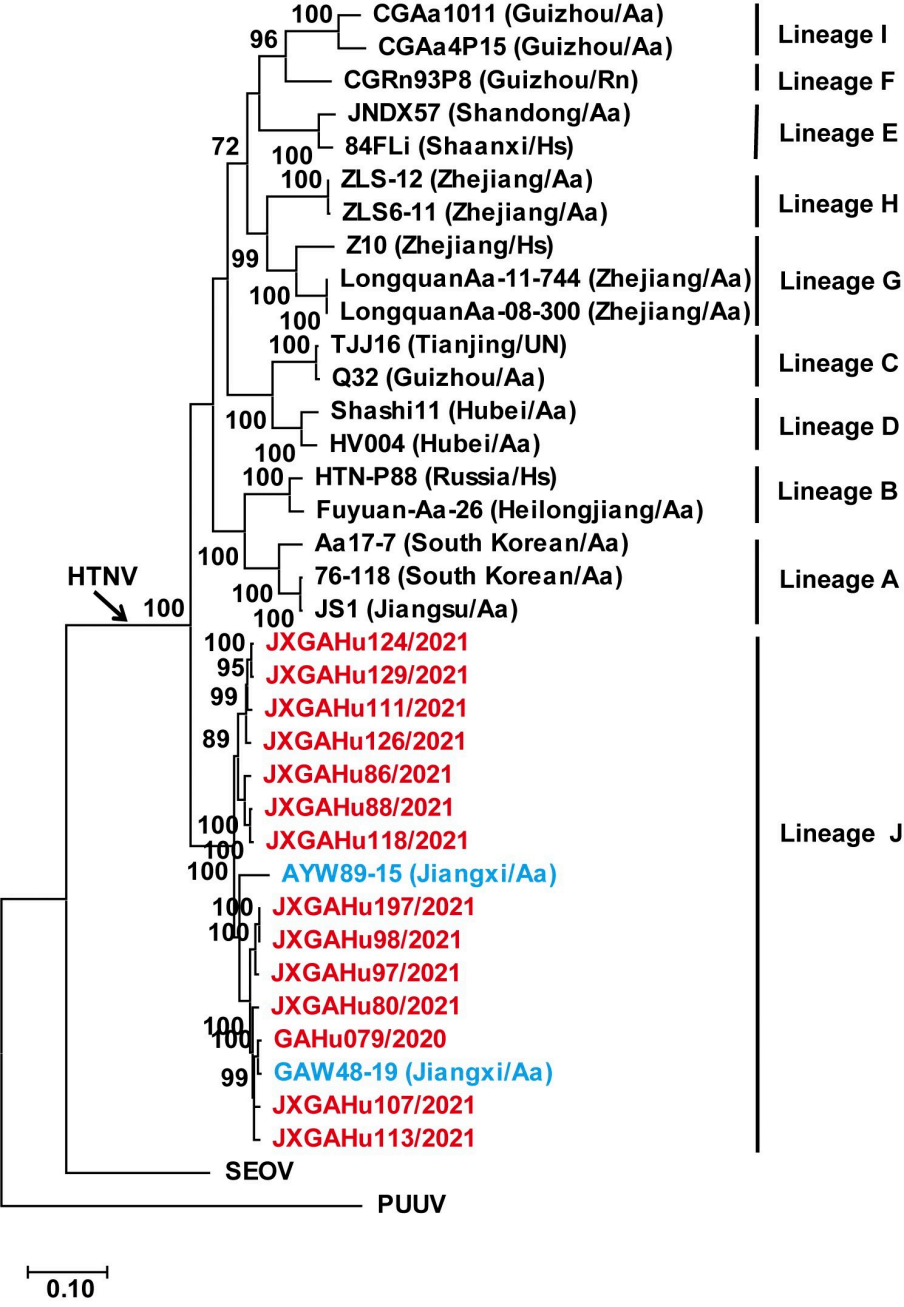
Phylogenetic tree of the orthohantavirus using the complete M sequence. The scale bars indicate the number of nucleotide substitutions per site. Strains labeled in red represent HTNVs from 14 patients in this study and those in blue represent HTNV isolates from rodents in Jiangxi but not in this study. PUUV, *Orthohantavirus puumalaense*. SEOV: *Orthohantavirus seoulense*.

The phylogenetic tree built based on the complete M segment is shown in **Fig 5,** and the phylogenetic trees constructed based on the complete S and L segments are shown in **S2 Fig.** The HTNVs in Jiangxi Province were distantly related to those from other areas. The HTNVs (including 238 strains) were classified as genetic Lineage A-J based on 5 group-specific amino acids on the NP, and 37 group-specific amino acids on the GP by Li et al, and the AYW89-15 strain from *A*. *agrarius* in Jiangxi was classified into the Lineage J, according to the group-specific amino acids L179 and D297 on NP, and K4, I241, E262, V327, I337, Y348, I664, G700 on GP [14]. The two group-specific amino acids on the NP of 14 HTNVs in this study were consistent with AYW89-15, and six (K4, I241, E262, V327, I337, G700) of the eight group-specific amino acids on the GP were consistent with AYW89-15. Based on the analysis of the phylogenetic tree and the group-specific amino acids, the 14 HTNVs in this study were classified into the new genetic lineage formed by AYW89-15 (Lineage J).

### Clinical characteristic

To know the clinical characteristics of HFRS caused by the HTNV genetic variants, the clinical symptoms and biological parameters of 53 patients were analyzed. As shown in **Table 2**, there were more men (62.3%, 33/53) than women, 62.2% (33/53) of patients were farmers and 83.0% (44/53) of patients were in rural settings. The median age was 37 years. The median duration of HFRS was 12 days. All patients (n = 53) had fever and thrombocytopenia. The severe shock was observed in 26.4% (14/53) of the cases. Some patients had gastrointestinal symptoms, such as diarrhea (18.9%) and nausea or vomiting (28.3%). However, pain (headache, myalgia, backache, and abdominal pain), petechiae, and respiratory symptoms were uncommon (**Table 2**).

**Table 2:** Characteristics of the 53 patients with HFRS identified by RT-qPCR, Jiangxi, 2020–2022.

Characteristics	Value
Males/total (%)	33/53 (62.3)
Median age, years [IQR]	37 [16–53]
Sample collection days post onset [IQR]	5 [4–6]
Duration of hospital stay [IQR]	12 [10–15]
Laboratory findings	
PLT, ×10^9^ /L [IQR] (NVR)	52 [34–71] (125–350)
PLT<125×10^9^/L (%)	53 (100%)
SCR, μmol/L [IQR, no. with missing data] (NVR)	94 [63–163, 8] (42–120)
SCR level >353.6 μmol/L (%)	7 (13.2%)
AST, U/L [IQR] (NVR)	107.2 [76.3–163.8] (15–40)
AST > ULN by more than 3-fold (%)	23 (43.4%)
AST > ULN by more than 5-fold (%)	11 (20.7%)
AST > ULN by more than 10-fold (%)	5 (9.4%)
LDH, U/L [IQR] (NVR)	546.4 [371.2–942.5] (114–240)
Symptoms (%):	
Fever (>39°C)	49 (92.5%)
Headache	10 (18.9%)
Myalgia	4 (7.5%)
Backache	4 (7.5%)
Abdominal pains	4 (7.5%)
Nausea or vomiting	15 (28.3%)
Diarrhea	10 (18.9%)
Cough, runny noses	4 (7.5%)
Petechiae in the conjunctiva	14 (26.4%)
Petechiae on skin	7 (13.2%)
Petechiae on the palate	11 (20.3%)
Hypotension (< 90/60 mmHg)	7 (13.2%)
Shock	14 (26.4%)

Abbreviations: IQR, interquartile range, PLT: platelet count, NVR: normal value rang.

**Fig 6:**
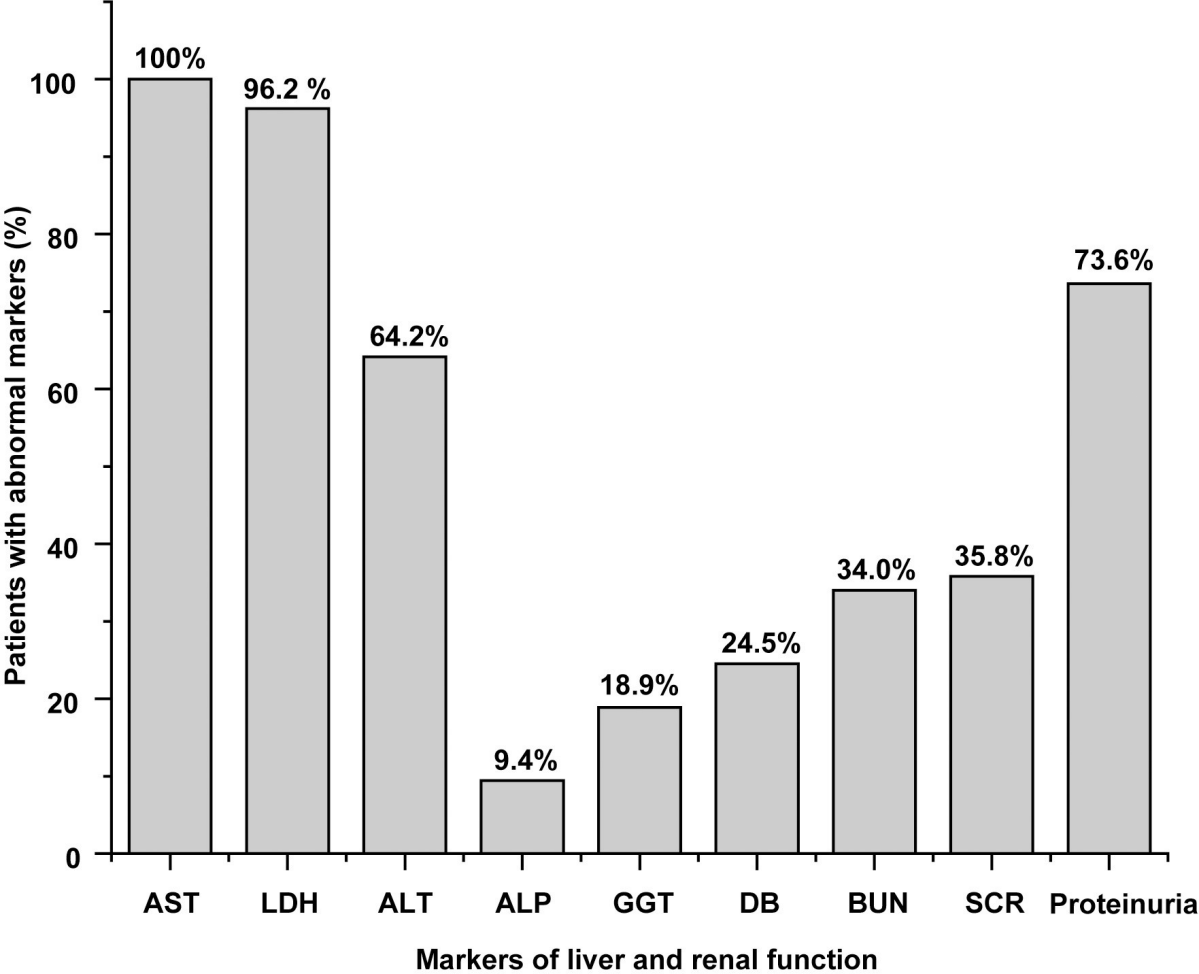
The proportion of patients with elevated liver and renal markers (n = 53). AST, LDH, ALT, ALP, GGT, and DB were liver function indicators, and proteinuria, BUN, and SCR were renal function indicators. The ULN levels of AST, LDH, ALT, ALP, GGT, DB, BUN, SCR, and proteinuria are 40 U/L, 240U/L, 50 U/L, 135 U/L, 60 U/L, 6 μmol/L, 120 μmol/L, 7.1 mmol/L, and 0.15 g/L, respectively.

Patients with HFRS in the acute phase showed common renal dysfunction as increased proteinuria, BUN and SCR can be found in 73.6%, 34.4%, and 35.8% of patients (**Fig 6**). Severe AKI (SCR level >353.6 μmol/L) was observed in 13.2% of cases. Abnormal liver function was common as elevated AST, LDH, and ALT were seen in 100%, 96.2%, and 64.2% of patients.

## Discussion

Genomic and viral isolation is crucial for identifying the etiological agents of infectious diseases. In the present study, samples were collected from patients with HFRS in the area where HTNV variants are found in rodents in Jiangxi province, China. Thirteen HTNVs were successfully isolated from the PBMCs of HFRS cases. The 13 isolates were identified through RT-qPCR, IFA, and whole gene sequencing. Whole genome sequences of HTNV were obtained from the 14 patients using amplicon-based NGS. Genetic analysis revealed that the sequence from the patients showed significant variations in nucleotide and amino acid to the HTNV strains found in other areas. A retrospective analysis was conducted on the clinical characteristics of the patients. This is the first report on the clinical characteristics of HFRS caused by HTNV variants in Jiangxi province, China. The results are important for the diagnosis of HFRS in clinical practice.

Orthohantaviruses are difficult to isolate in vitro, particularly from clinical samples such as serum and plasma [27]. As serum and plasma were the common clinical samples used for orthohantanviral isolation, in the early stages of this study 21 plasma samples from clinical cases were used for viral isolation in Vero-E6 culture but no isolates were recovered (**Fig 2**). Studies revealed that orthohantaviruses could infect immune cells such as macrophages and mononuclear blood cells originating from PBMC [1,12,28]. Early research successfully isolated the hantavirus strain B-1 from peripheral blood cells, and the virus can be grown in mononuclear cells [29]. Viruses have been successfully recovered from PBMC in several human viral infections, including polio, rubella, measles, adenovirus pneumonia, and dengue viruses [30]. These suggest that PBMC of patients with HFRS may carry infectious orthohantaviruses, which may be useful for viral isolation. The low recovery rate of orthohantavirus from plasma prompted us to investigate the potential of obtaining the virus from PBMCs. Therefore, plasma and paired PBMC from 7 confirmed HTNV cases were inoculated to Vero-E6 cells, 3 HTNV isolates were recovered from PBMCs, and none was recovered from plasma. Finally, 13 isolates were recovered from 32 PBMCs, with a recovery rate of > 40%. Our results showed that PBMCs were particularly efficient samples in isolating orthohantaviruses from patients with HFRS. These results provide useful guidance for orthohantaviruses isolation from clinical cases.

High-throughput sequencing technology provides a robust platform for studying genetic diversity within viral populations [5]. Wang et al. revealed that a minimum sequencing depth of 400× and 1000× is required to identify variants at 1% and 0.5% levels with 99.999% confidence in viruses [31]. Increasing sequencing depth would improve sensitivity to meet these requirements. Amplicon-based NGS is sensitive to achieving complete genome coverage and generating great read depth. A critical step for using amplicon-based sequencing is the availability of obtaining close and reliable reference sequences, and primers for amplicon need regular updating according to viral mutation [32]. In this study, the primers are designed based on the gene-conserved domain of the GAW48-19 strain with application lengths of 1000 to 1700 bases. The portion of mapped reads in this study to the reference sequences was 32.5–98.3%. Nearly full genome coverage of HTNV (99.8–100%) with average depths of the S, M, and L segments were 33,768, 37,881, and 53,063, respectively. Our data suggest that the amplicon-based method is suitable for obtaining high-quality whole-genome sequences of HTNV in the NGS platform.

The complete genetic analysis showed significant differences in nucleotide and amino acid levels between the HTNVs from the patients in this study and the HTNV strains from other regions. Four specific amino acids related to 6 immune epitopes on NP and GP of HTNVs were found. Phylogenetic analyses revealed that all HTNVs from 14 patients belong to a new genetic lineage (Lineage J). These results of viral isolation and complete gene analysis suggested that the HTNV variant caused human infection. In addition to genetic variation, gene recombination, and reassortment are important evolutionary mechanisms of segmented RNA viruses [33]. Recombination has been observed in the M segments of HTNV and S segments of the Tula virus [14,34]. In our study, RDP5 detected a recombination event in the L segment of JXGAHu197/2021. These findings show the diversity of orthohantavirus recombination. The recombinant viruses may lead to different disease outcomes. It is worth monitoring the prevalence of recombinant strains and identifying potential changes in the virulence and replication ability.

Fever, headache, myalgia, back pain, and petechiae are the characteristic symptoms of HFRS in the early stage [9,35,36]. A study conducted in Korea reported that fever, headache, myalgia, back pain, and petechiae were observed in 100%, 84%, 75%, 93%, and 94% of patients infected with HTNV, respectively [36]. A study in China found 100%, 72.5%, 68.8%, 80%, and 56.3% of fever, headache, myalgia, back pain, and petechiae, respectively, in patients with HTNV infection [37]. In this study, all patients had fever, which is consistent with previous studies. However, symptoms such as headache (7.5%), myalgia (7.5%), back pain (7.5%), and petechiae (total of 35.8%) were less common than reported in previous studies [36–38]. Gastrointestinal symptoms such as abdominal pain were 45–85% in other studies [36–39], but the frequency of abdominal pain (7.5%) was considerably lower in this study. These results indicate atypical clinical symptoms of HFRS caused by the HTNV variants in Jiangxi Province, China. It is difficult to diagnose HFRS infection based on the clinical symptoms in this area, and laboratory diagnoses are needed.

According to the laboratory test results, abnormal parameters, including PLT (100%), AST (100%), LDH (96.2%), and proteinuria (73.6%) were common. Therefore, patients who have a recent (60 days prior) travel history or reside in this area, present with high fever (>39°C), thrombocytopenia, proteinuria, increase in AST and LDH, and an orthohantavirus infection should be considered. Serological IgM testing is commonly used for specific diagnoses of orthohantavirus infection during the acute phase. However, RT-qPCR seems more sensitive and specific. RT-qPCR confirmed the diagnosis in 9.6% of patients who initially tested negative for specific PUUV antibodies early in the disease [1,40].

Renal dysfunction was observed as increased SCR (35.8%), BUN (34.0%), and proteinuria (73.6%) in this study. Severe AKI (SCR level >353.6 μmol/L) was observed in 13.2% of cases. In addition to renal dysfunction, attention should be paid to the common liver injuries caused by these variants. The frequency of increased AST (100%) and the median value (107.2 U/L) in this study were considerably higher than that (67.6%, 63 U/L) in the previous report [36]. The activities of AST 1-fold to 10-fold higher than the ULN were present in 100% to 9.4% of patients, respectively, indicating that the HTNV variants were associated with mild to severe liver damage. The pathogenic mechanism of the HTNV variants required further investigation.

This study has some limitations: First, the clinical characteristics were analyzed during the early stage of the disease, and attention needs to be paid to the clinical progress in the later stages to understand the clinical characteristics comprehensively. Second, this study lacked information on patient medications before hospitalization and could not analyze the impact of medication on liver function.

## Conclusions

Viral isolation and whole genome sequencing confirm that the HTNV variant causes human infection, in Jiangxi province. PBMCs are particularly efficient samples in isolating orthohantaviruses from patients with HFRS. The clinical symptoms of HFRS caused by HTNV variants during the acute phase are atypical, and laboratory diagnoses are needed. In addition to renal dysfunction, liver injuries are commonly caused by these variants. These results provide useful guidance for orthohantaviruses isolation from clinical cases and are important for HFRS diagnosis in clinical practice.

## Supporting information

S1 Table Primers for one-step amplicon-based NGS. (XLSX)

S2 Table Reference sequences of orthohantavirus were used for analysis in this study. (XLSX)

S3 Table Detailed nucleotide identity and amino acid similarity between the isolates from patients and GAW48-19 from *A. agrarius*. (XLSX)

S4 Table The specific amino acid mutations in epitopes of HTNVs in Jiangxi province, China. (XLSX)

S5 Table The recombinant event in the L segment in JXGAHu197/2021. (XLSX)

S1 Fig TEM images of HTNV strain (strain JXGAHu98/2021) particles by negative stain. HTNV particles were shown by the arrow. The supernatant of JXGAHu98/2021 was condensed at a ratio of 1:200 by centrifugation using a 100 kDa ultrafiltration tube. The condense was applied to holey electron microscopy (EM) grids, negatively stained, and observed at 200KVwith a Tecnai G^2^ 20 Twin electron microscope.
(TIF)

S2 Fig Phylogenetic trees of the orthohantavirus using the complete S and L sequences. The scale bars indicate the number of nucleotide substitutions per site. Strains labeled in red represent HTNVs from 14 patients in this study including 13 isolated strains from Vero-E6 cells and one strain (GAHu79/2020) from a plasma sample, and those in blue represent HTNV strains previously isolated from rodents in Jiangxi but not in this study. PUUV, *Orthohantavirus puumalaense*.
(TIF)
