# Supplementary figures and images for: Isolation and characterization of genetic variants of Orthohantavirus hantanense from clinical cases of HFRS in Jiangxi Province, China
Source: PLoS Negl Trop Dis. 2024 Sep 5;18(9):e0012439. doi: 10.1371/journal.pntd.0012439 (PMC11376573; doi:10.1371/journal.pntd.0012439)

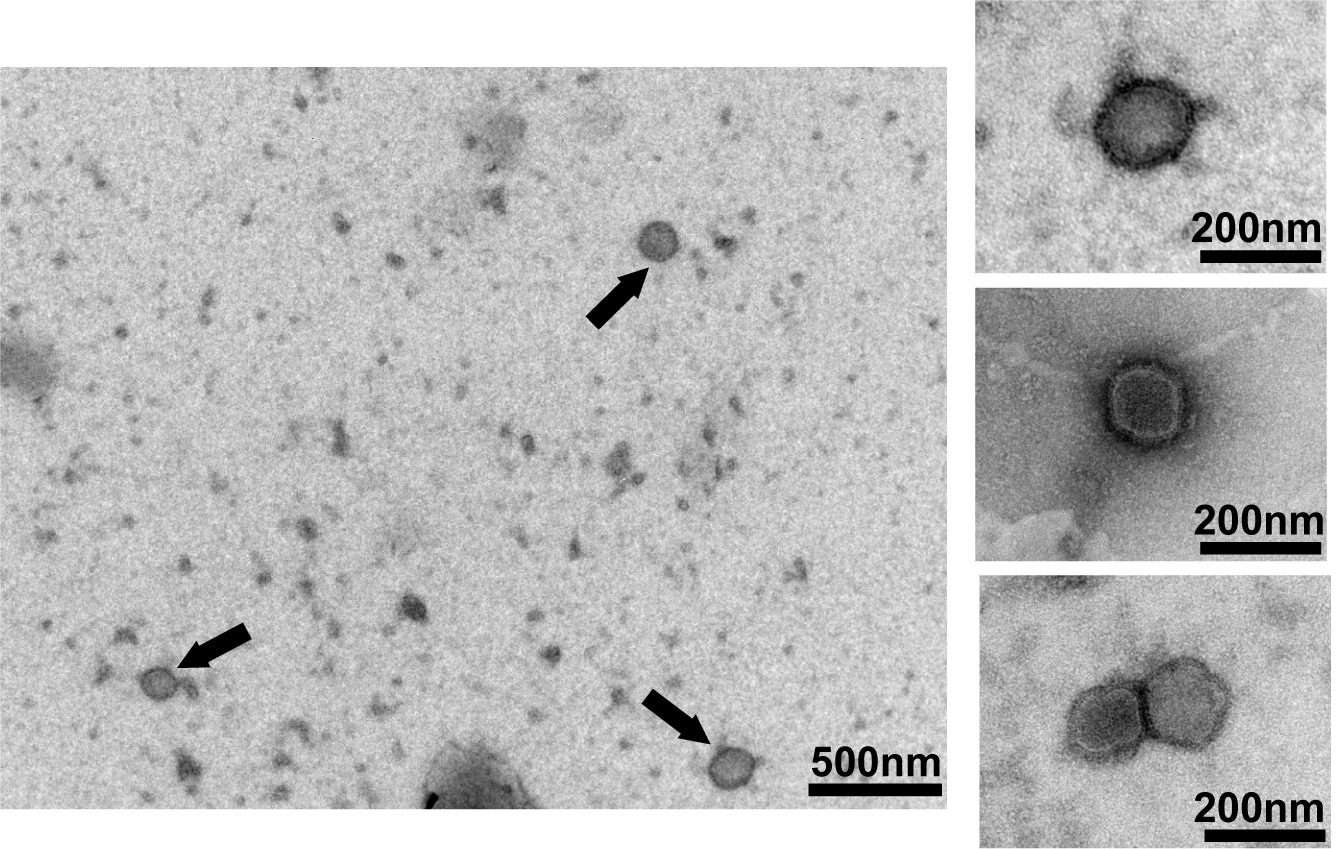

Supplement: S1 Fig — HTNV particles were shown by the arrow. The supernatant of JXGAHu98/2021 was condensed at a ratio of 1:200 by centrifugation using a 100 kDa ultrafiltration tube. The condense was applied to holey electron microscopy (EM) grids, negatively stained, and observed at 200KVwith a Tecnai G2 20 Twin electron microscope. (TIF) [file pntd.0012439.s006.tif]

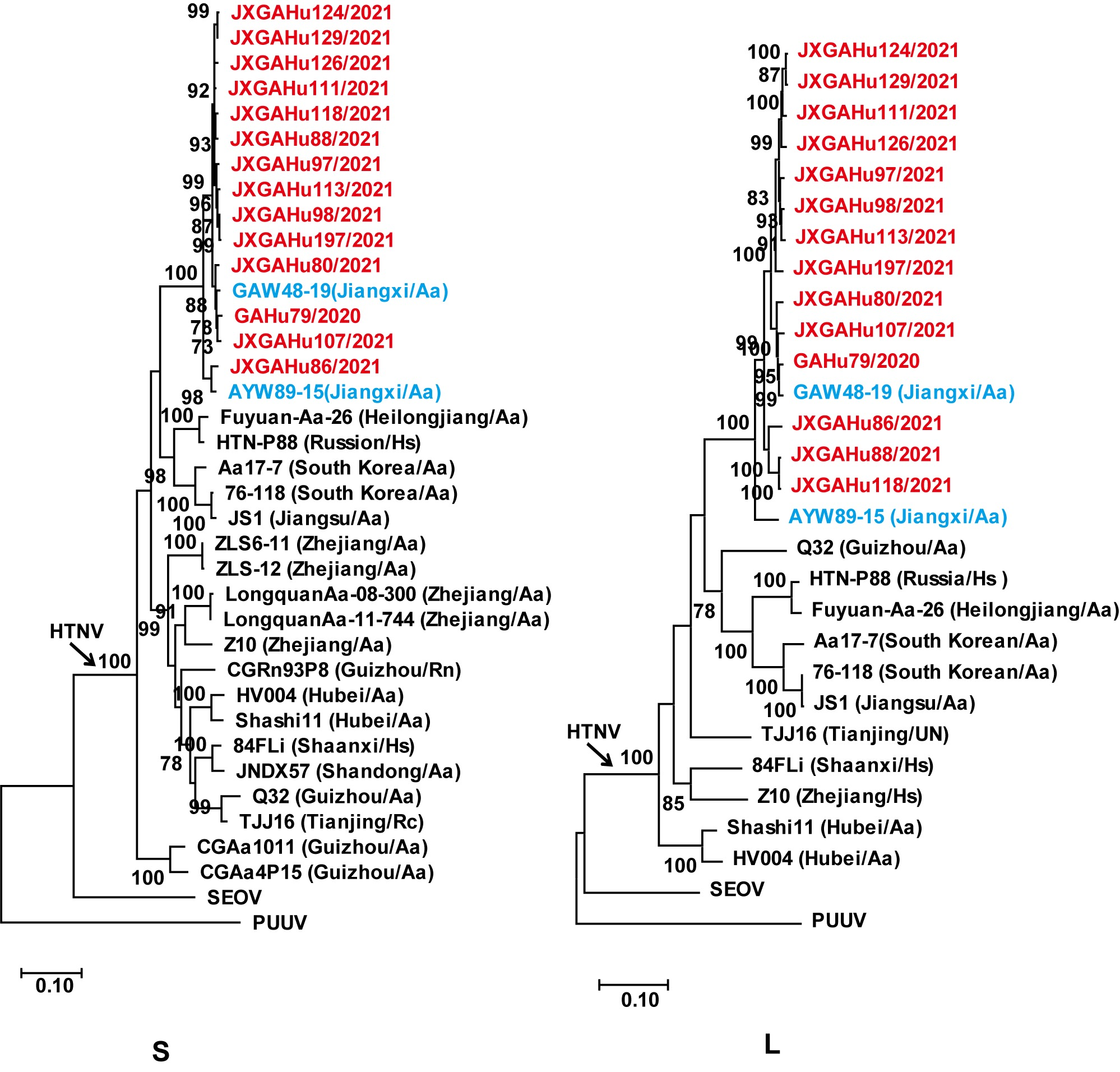

Supplement: S2 Fig — The scale bars indicate the number of nucleotide substitutions per site. Strains labeled in red represent HTNVs from 14 patients in this study including 13 isolated strains from Vero-E6 cells and one strain (GAHu79/2020) from a plasma sample, and those in blue represent HTNV strains previously isolated from rodents in Jiangxi but not in this study. PUUV, Orthohantavirus puumalaense. (TIF) [file pntd.0012439.s007.tif]
